# Supplementary material for: HIV-associated gut dysbiosis is independent of sexual practice and correlates with noncommunicable diseases
Source: Nat Commun. 2020 May 15;11:2448. doi: 10.1038/s41467-020-16222-8 (PMC7228978; doi:10.1038/s41467-020-16222-8)
Supplement: Supplementary file 1 — Supplementary Information [file 41467_2020_16222_MOESM1_ESM.pdf]

Supplementary Information

HIV infection-associated gut dysbiosis is present independently of sexual practice and correlates with noncommunicable diseases

Vujkovic-Cvijin, Sortino *et al.*

Supplementary Figures

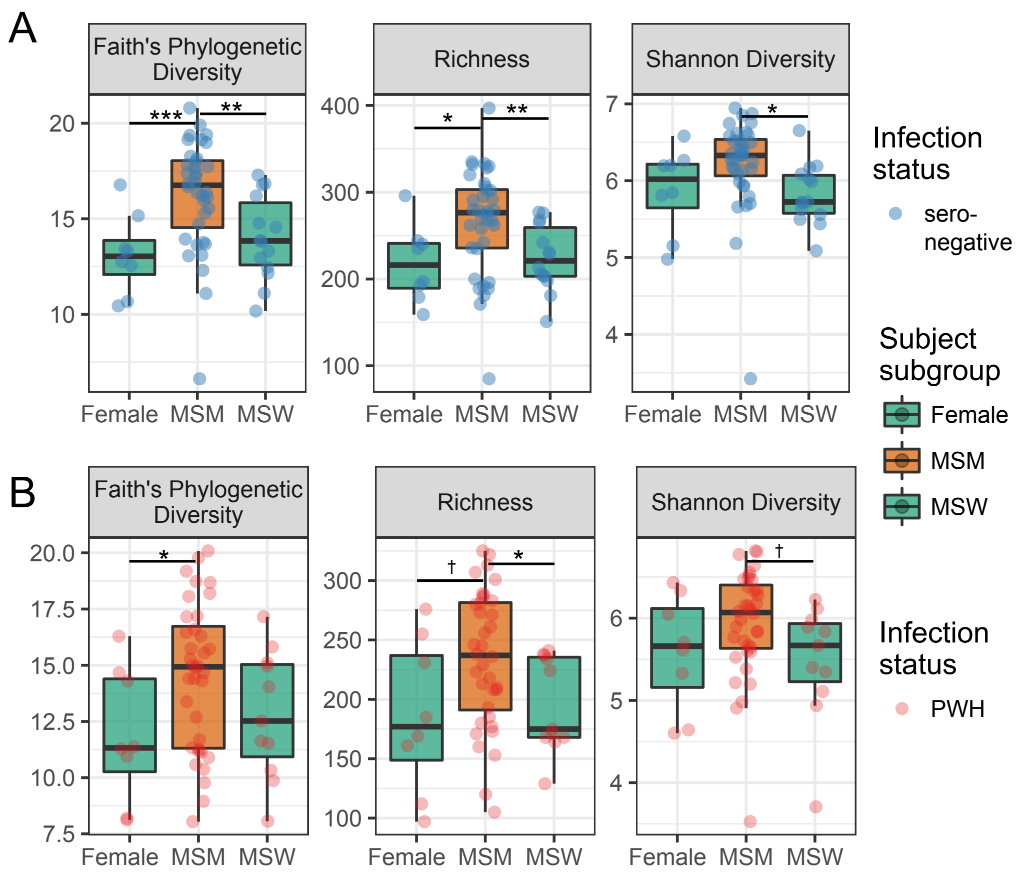

Supplementary Figure 1. Alpha diversity shifts by sex/sexual practice subject groupings. Boxes denote inter-quartile range, bar denotes median, and whiskers denote range. A) Alpha diversity measures among HIV-seronegative subjects by each subject grouping (female n=8, MSM n=36, MSW n=14). Faith's Phylogenetic Diversity P=0.0017, Richness P=0.0005, Shannon Diversity P=0.0031 by Kruskal-Wallis tests. B) Alpha diversity measures among PWH subjects by each subject grouping (female n=8, MSM n=36, MSW n=11). Faith's Phylogenetic Diversity

$P=3.4*10^{-5}$, Richness P=0.0002, Shannon Diversity P=0.0002 by Kruskal-Wallis tests. †P<0.10, *P<0.05, **P<0.005, ***P<0.001 by two-tailed Mann-Whitney U tests.

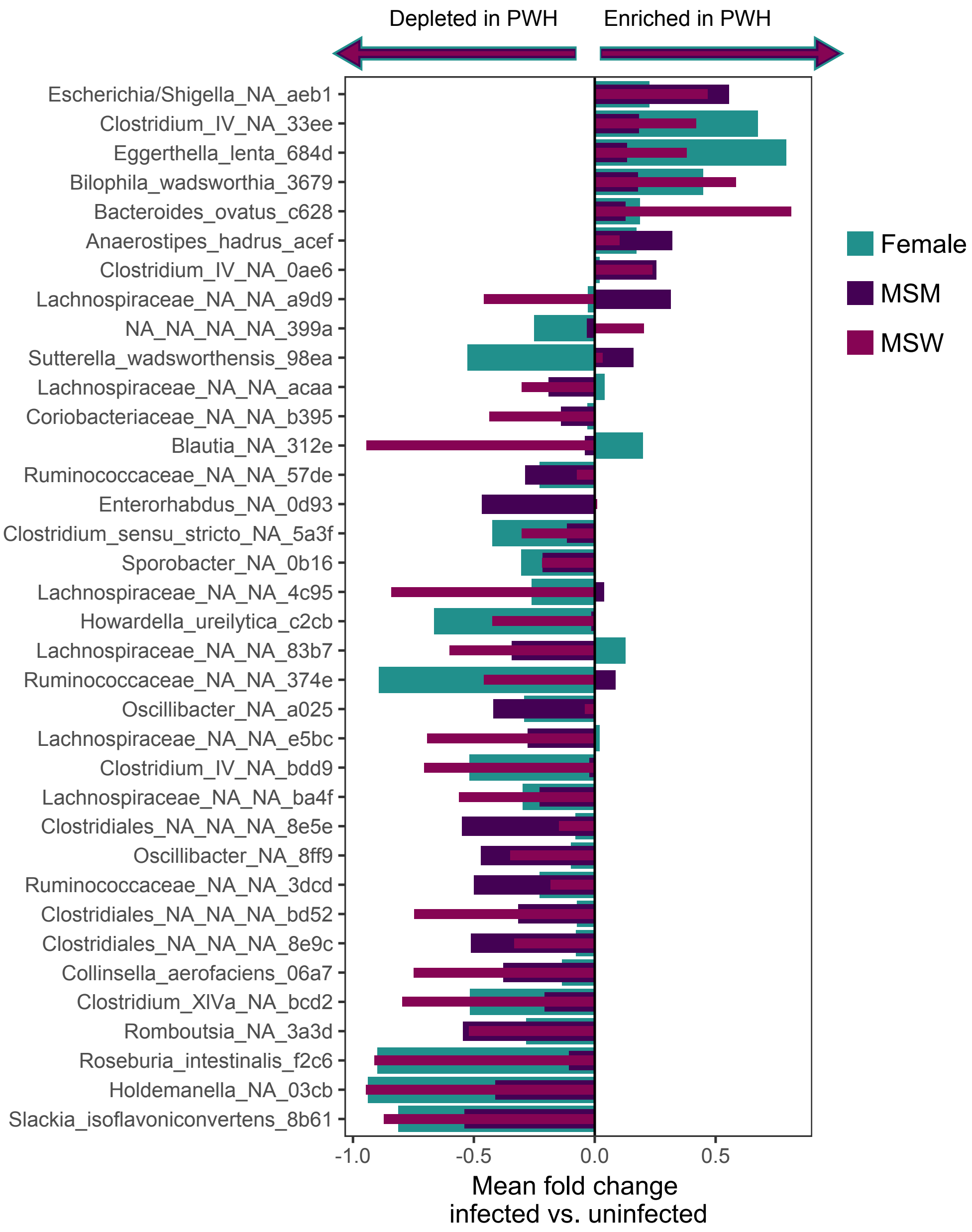

Supplementary Figure 2. Bacterial taxa shifts within each subject group exhibit common patterns. Paired Wilcoxon tests were performed within each sex/sexual practice subject grouping for each ASV. The top 15 ASVs for each subject grouping were selected, and their abundance shifts (log mean fold change in PWH vs uninfected) for each grouping are shown. Fisher's exact test $P=9*10^{-4}$.

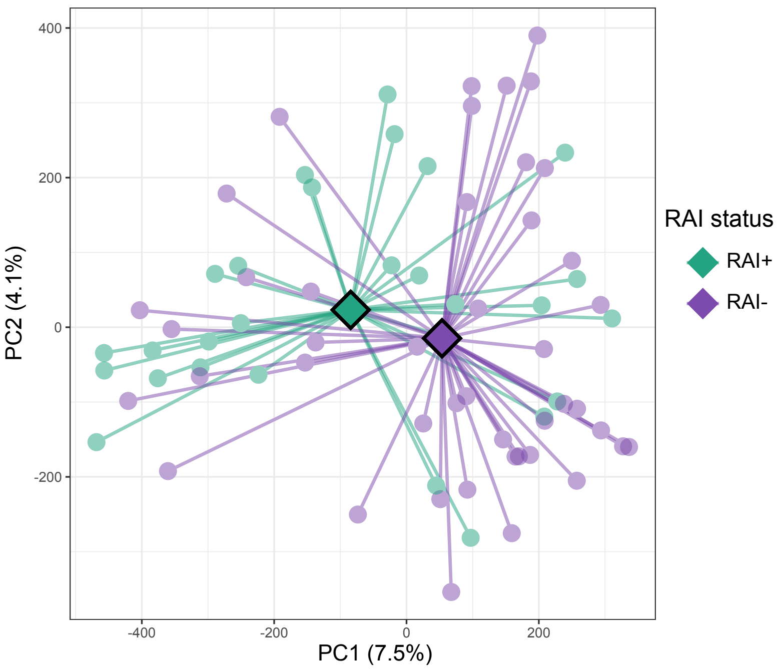

Supplementary Figure 3. Receptive anal intercourse within six months prior to sampling (RAI) segregates MSM subject microbiota profiles. PCoA plot of MSM subjects that reported either RAI or no RAI is shown to cluster subjects (PERMANOVA P=0.007).

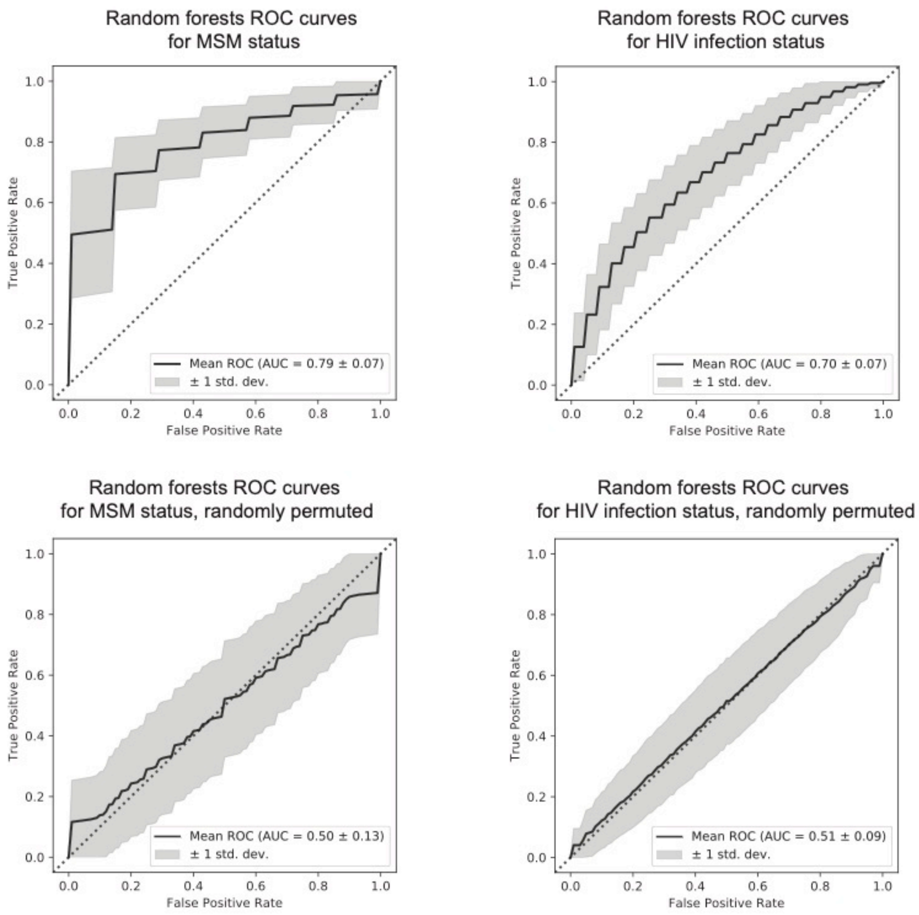

Supplementary Figure 4. Random forests machine learning analysis mirrors that of ridge regression machine learning. Analyses analogous to those in Figure 5 were performed using random forests, and ROC curves are shown for each comparison (MSM vs. MSW [n=72 MSM, n=22 MSW], and PWH vs. uninfected [n=71 PWH, n=71 seronegative]). Sample classifications (e.g. MSM, MSW, PWH, seronegative) were randomly permuted to introduce noise similarly as in Figure 5. Subject samples included in these analyses and those of Figure 5 are described in detail in Methods.

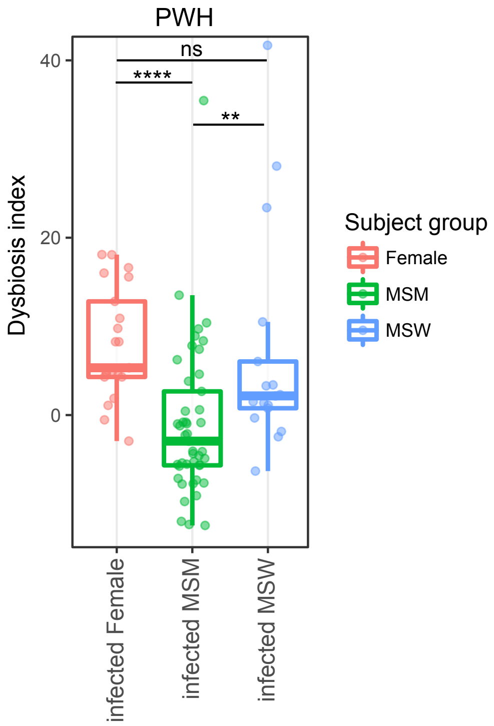

Supplementary Figure 5. Dysbiosis index (DI) distribution across all PWH split by subject groups (female, MSM, MSW). MSM exhibit lower DI than females and MSW. **P<0.005, ****P<0.0001, unpaired T-test. P=6*10^{-6} for MSM vs. female, P=0.0034 for MSM vs. MSW by unpaired two-sided Mann-Whitney U test. Female n=19, MSM n=44, MSW n=17. Boxes denote inter-quartile range, bar denotes median, and whiskers denote range.

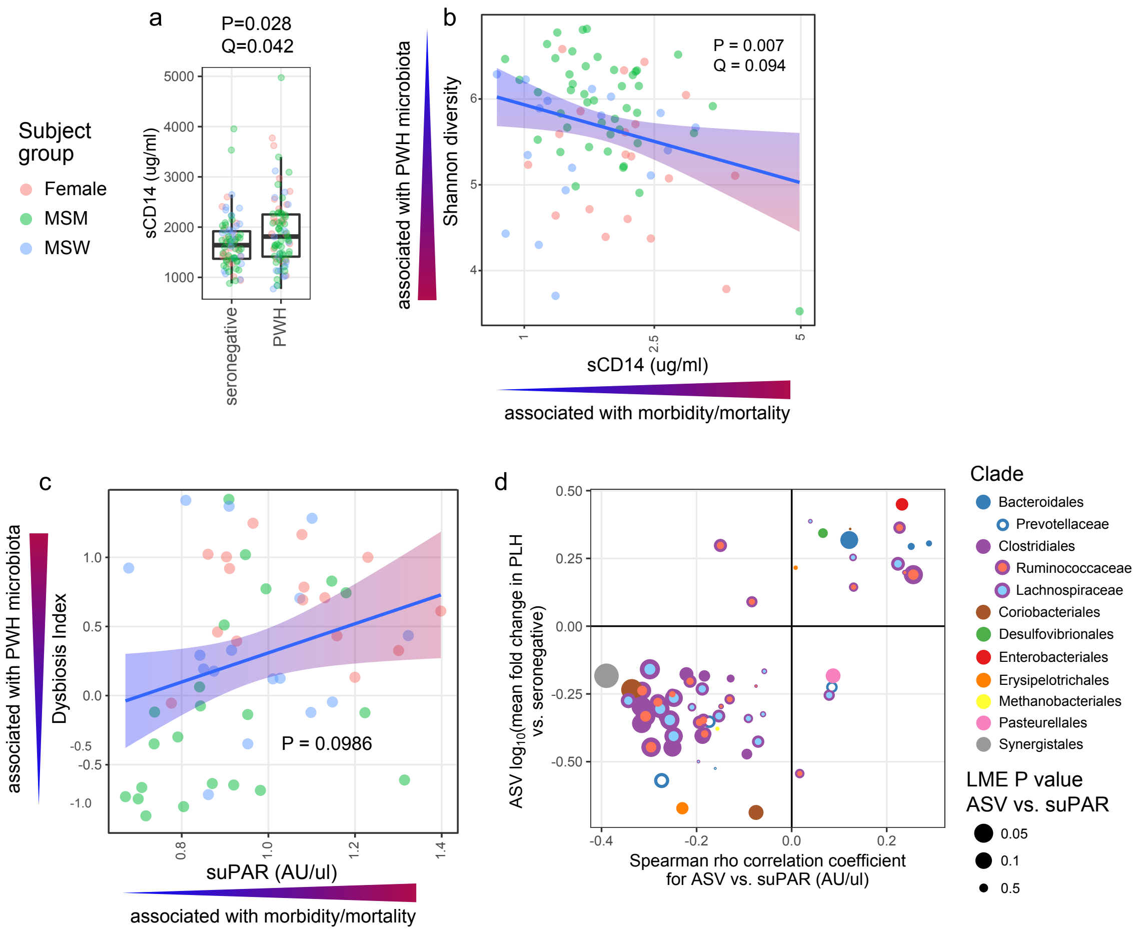

Supplementary Figure 6. Associations between inflammatory markers of HIV infection and microbiota features. For all analyses, P value was calculated by linear mixed effects models (LME) with subject group as a random effect, and Q values were calculated by the Benjamini-Hochberg method. A) sCD14 is enriched in PWH (n=80) compared to seronegative controls (n=80) by LME, concordant with prior studies. Boxes denote inter-quartile range, bar denotes median, and whiskers denote range. B) Shannon diversity correlates with sCD14 among PWH (n=80 PWH) by LME. C) The Dysbiosis Index correlates weakly with suPAR (P=0.0986, by LME, n=54 PWH). D) ASVs of the Dysbiosis Index exhibit enrichment patterns consistent with positive correlation with suPAR, and depletion patterns consistent with negative correlations with suPAR. Each point represents one ASV that was found to differ in abundance

between PWH and seronegative controls (having two-tailed Wilcoxon $P < 0.05$ in PWH vs. seronegative, as defined for taxa that comprise the DI). Fold enrichment in PWH vs seronegative represents the y-axis, while Spearman rho correlation coefficients for the direction of association between each ASV and suPAR concentrations represents the x-axis. Point size reflects the strength of the association between ASV relative abundance and suPAR concentration by linear mixed effects, with subject group as a random effect.

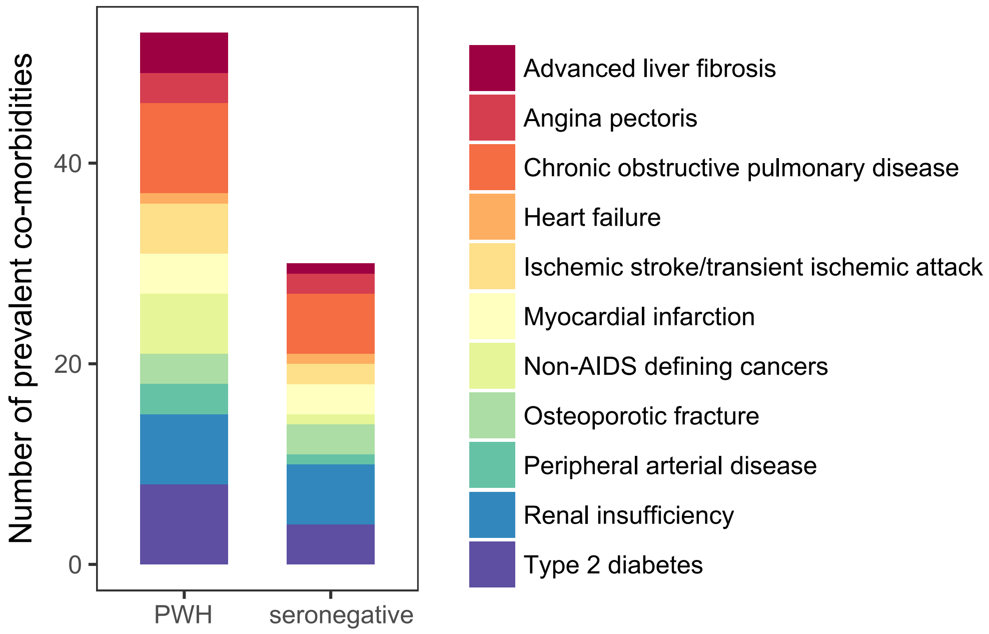

Supplementary Figure 7. Distribution of comorbidities among PWH and seronegative subjects. Shown are numbers of prevalent noncommunicable age-associated comorbidity that occurred within all PWH subjects and seronegative subjects, respectively, until the date of fecal sampling.

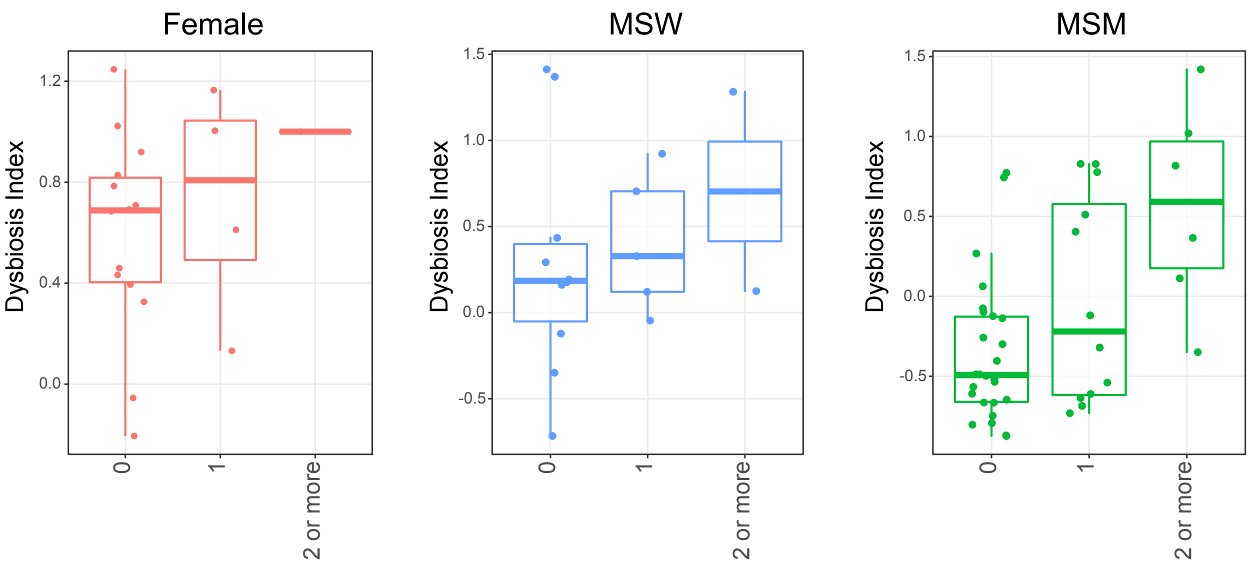

Supplementary Figure 8. Prevalence of comorbidities associates with DI irrespective of subject grouping. DI among subjects split by subject grouping (F, MSM, MSW) and number of comorbidities experienced. Comparisons among MSM showed a statistically significant association between DI and comorbidity number (P = 0.0073, rho = 0.4 by Spearman correlation test), while results did not reach significance for females and MSW. Boxes denote inter-quartile range, bar denotes median, and whiskers denote range.

Supplementary Note 1:

AGE$_{h}$IV Cohort Study Group Members:

M. van der Valk[1,6], J. Schouten[1,13], K.W. Kooij[1], R.A. van Zoest[1], S.O. Verboeket[1], M.L.Verburgh, B.C. Elsenga[1] , M. Prins[2], L. del Grande[2], V. Olthof [2], I. Agard[2], S. Zaheri[3], M.M.J. Hillebregt[3], Y.M.C. Ruijs[3], D.P. Benschop[3], A. el Berkaoui[3], A.M. Harskamp-Holwerda[4], I. Maurer[4], M.M. Mangas Ruiz[4], A.F. Girigorie[4], B. Boeser-Nunnink[4], W. Zikkenheiner[5], F.R. Janssen[5], S.E. Geerlings[6], A. Goorhuis[6], J.W.R. Hovius6, F.J.B. Nellen6, T. van der Poll[6], J.M. Prins[6], W.J. Wiersinga[6], M. van Vugt[6], G. de Bree[6], J. van Eden[6], A.M.H. van Hes[6], F.J.J. Pijnappel[6], A. Weijsenfeld[6], S. Smalhout[6], M. van Duinen[6], A. Hazenberg[6], P.G. Postema[7], P.H.L.T. Bisschop[8], M.J.M. Serlie[8], P. Lips[9], E. Dekker[10], N. van der Velde[11], J.M.R. Willemsen[12], L. Vogt[12], P. Portegies, B.A. Schmand[13], G.J. Geurtsen[13], F.D. Verbraak[14], N. Demirkaya[14], I. Visser[15], A. Schadé[15], P.T. Nieuwkerk[16], N. Langebeek[16], R.P. van Steenwijk[17], E. Dijkers[17], C.B.L.M. Majoie[18], M.W.A. Caan[18], H.W. van Lunsen[19], M.A.F. Nievaard[19], B.J.H. van den Born[20], E.S.G. Stroes[20], W.M.C. Mulder[21], S. van Oorspronk[21]

Affiliations

1. Amsterdam University Medical Centers (Amsterdam UMC), University of Amsterdam, Department of Global Health and Amsterdam Institute for Global Health and Development (AIGHD).

2. Public Health Service of Amsterdam, Department of Infectious Diseases.

3. HIV Monitoring Foundation.

4. Amsterdam UMC, Laboratory for Viral Immune Pathogenesis and Department of Experimental Immunology.

5. AIGHD

6. Amsterdam UMC, Division of Infectious Diseases

7. Amsterdam UMC, Department of Cardiology

8. Amsterdam UMC, Division of Endocrinology and Metabolism

9. Amsterdam UMC

10. Amsterdam UMC, Department of Gastroenterology

11. Amsterdam UMC, Division of Geriatric Medicine

12. Amsterdam UMC, Division of Nephrology

13. Amsterdam UMC, Department of Neurology

14. Amsterdam UMC, Department of Ophthalmology

15. Amsterdam UMC, Department of Psychiatry

16. Amsterdam UMC, Department of Medical Psychology

17. Amsterdam UMC, Department of Pulmonary medicine

18. Amsterdam UMC, Department of Radiology

19. Amsterdam UMC, Department of Gynaecology

20. Amsterdam UMC, Division of Vascular Medicine

21. HIV Vereniging Nederland
