# Supplementary material for: HIV-associated gut dysbiosis is independent of sexual practice and correlates with noncommunicable diseases
Source: Nat Commun. 2020 May 15;11:2448. doi: 10.1038/s41467-020-16222-8 (PMC7228978; doi:10.1038/s41467-020-16222-8)
Supplement: Supplementary file 3 — Description of Additional Supplementary Files [file 41467_2020_16222_MOESM3_ESM.pdf]

Description of Additional Supplementary Files

File name: Supplementary Data 1
Description: Cohort characteristics stratified by subgroup (female, MSM, MSW) and HIV infection status.

File name: Supplementary Data 2
Description: Immunologic and virological parameters of PWH in cohort.

File name: Supplementary Data 3
Description: Factors that associate with community compositional variance.

File name: Supplementary Data 4
Description: Taxa that differenitate PLWH vs. seronegative, paired Wilcoxon tests.

File name: Supplementary Data 5
Description: Taxa that differenitate MSM vs. MSW, unpaired Mann-Whitney tests.

File name: Supplementary Data 6
Description: Taxa that differenitate MSM/RAI+ vs MSM/RAI-, unpaired Mann-Whitney tests.

File name: Supplementary Data 7
Description: Ridge logistic regression feature coefficients in comparison of MSM vs MSW.

File name: Supplementary Data 8
Description: Ridge logistic regression feature coefficients in comparison of PLWH vs seronegative.

File name: Supplementary Data 9
Description: Variables that associate with Dysbiosis Index using linear mixed effects to control for subject group.

File name: Supplementary Data 10
Description: Variables that associate with Shannon diversity using linear mixed effects to control for subject group.

File name: Supplementary Data 11
Description: Associations among PWH subjects between aptamer-based protein quantifications and HIV infection, Shannon diversity, and the Dysbiosis Index (DI).
