# Supplementary material for: HIV-associated gut dysbiosis is independent of sexual practice and correlates with noncommunicable diseases
Source: Nat Commun. 2020 May 15;11:2448. doi: 10.1038/s41467-020-16222-8 (PMC7228978; doi:10.1038/s41467-020-16222-8)
Supplement: Supplementary file 15 — Reporting Summary [file 41467_2020_16222_MOESM15_ESM.pdf]

nature research

Corresponding author(s): Irini Sereti

Last updated by author(s): Mar 20, 2020

Reporting Summary

Nature Research wishes to improve the reproducibility of the work that we publish. This form provides structure for consistency and transparency in reporting. For further information on Nature Research policies, see Authors & Referees and the Editorial Policy Checklist .

Statistics

For all statistical analyses, confirm that the following items are present in the figure legend, table legend, main text, or Methods section.

n/a	Confirmed	
☐	☒	The exact sample size (*n*) for each experimental group/condition, given as a discrete number and unit of measurement
☐	☒	A statement on whether measurements were taken from distinct samples or whether the same sample was measured repeatedly
☐	☒	The statistical test(s) used AND whether they are one- or two-sided<br>*Only common tests should be described solely by name; describe more complex techniques in the Methods section.*
☐	☒	A description of all covariates tested
☐	☒	A description of any assumptions or corrections, such as tests of normality and adjustment for multiple comparisons
☐	☒	A full description of the statistical parameters including central tendency (e.g. means) or other basic estimates (e.g. regression coefficient) AND variation (e.g. standard deviation) or associated estimates of uncertainty (e.g. confidence intervals)
☐	☒	For null hypothesis testing, the test statistic (e.g. *F*, *t*, *r*) with confidence intervals, effect sizes, degrees of freedom and *P* value noted<br>*Give P values as exact values whenever suitable.*
☐	☒	For Bayesian analysis, information on the choice of priors and Markov chain Monte Carlo settings
☐	☒	For hierarchical and complex designs, identification of the appropriate level for tests and full reporting of outcomes
☐	☒	Estimates of effect sizes (e.g. Cohen's *d*, Pearson's *r*), indicating how they were calculated

Our web collection on statistics for biologists contains articles on many of the points above.

Software and code

Policy information about availability of computer code

Data collection	STATA v12.0 and Limesurvey v2.6.7 were used for data collection and curation.
Data analysis	All analyses were performed as described in methods using R (v3.5.2) packages ape (v5.3), dada2 (v1.10.1), digest (v0.6.23), dplyr (v0.8.3), exactRankTests (v0.8.30), geepack (v1.3.1), ggplot2 (v3.2.1), ggthemes (v4.2.0), grid (v3.5.3), lme4 (v1.1.21), lmerTest (v3.1.0), plyr (v1.8.4), RColorBrewer (v1.1.2), reshape2 (v1.4.3), splitstackshape (v1.4.8), vegan (v2.5.6), viridis (v0.5.1). Additionally, the terminal suite QIIME2 (v2018.8) was used for alpha diversity assessments.

For manuscripts utilizing custom algorithms or software that are central to the research but not yet described in published literature, software must be made available to editors/reviewers. We strongly encourage code deposition in a community repository (e.g. GitHub). See the Nature Research guidelines for submitting code & software for further information.

Data

Policy information about availability of data

All manuscripts must include a data availability statement. This statement should provide the following information, where applicable:

- Accession codes, unique identifiers, or web links for publicly available datasets
- A list of figures that have associated raw data
- A description of any restrictions on data availability

All 16s microbiota profiling data, used to generate all figures, were derived from Illumina sequencing raw data available at PRJNA589036 (BioProject) and SRP229524 (SRA). Normalized data for proteomic screens (Supplementary Table 11, Figure 6, Supplementary Figure 6) can be found in Source Data.

Field-specific reporting

Please select the one below that is the best fit for your research. If you are not sure, read the appropriate sections before making your selection.

☒ Life sciences ☐ Behavioural & social sciences ☐ Ecological, evolutionary & environmental sciences

For a reference copy of the document with all sections, see nature.com/documents/nr-reporting-summary-flat.pdf

Life sciences study design

All studies must disclose on these points even when the disclosure is negative.

Sample size	Sample sizes were not devised based on power calculations; our limitations were primarily numbers of paired samples that could be identified that were matched for various host variables as described in the main text. We profiled all available samples that met matching criteria. Thus, all available samples were profiled without pre-determined sample size minima.
Data exclusions	For paired analyses, samples for whom the paired sample was missing were excluded from analysis (n=18). This occurred due to a variety of reasons including failure to PCR amplify or insufficient reads obtained.
Replication	We replicated results via usage of multiple different analyses. PERMANOVA and random forests are two conceptually unrelated methods of testing the robustness of microbiota community differences between subject groups, and were both used. All such analyses are presented in the manuscript (Figures 1, 2, 5).
Randomization	We randomized DNA extraction of stool samples across two plates by forcing a balanced representation of PLWH and seronegative subjects, as well as subject groups, on each stool extraction plate.
Blinding	We were not blinded to HIV serostatus or subject group, as these were critical for proper analysis.

Reporting for specific materials, systems and methods

We require information from authors about some types of materials, experimental systems and methods used in many studies. Here, indicate whether each material, system or method listed is relevant to your study. If you are not sure if a list item applies to your research, read the appropriate section before selecting a response.

Materials & experimental systems

n/a	Involved in the study	
☒	☐	Antibodies
☒	☐	Eukaryotic cell lines
☒	☐	Palaeontology
☒	☐	Animals and other organisms
☐	☒	Human research participants
☐	☒	Clinical data

Methods

n/a	Involved in the study	
☒	☐	ChIP-seq
☒	☐	Flow cytometry
☒	☐	MRI-based neuroimaging

Human research participants

Policy information about studies involving human research participants

Population characteristics	A cohort of matched PWH and HIV-seronegative controls (total n=160, PWH n=80, HIV-seronegative controls n=80), was assembled to include stratified subgroups of 83 MSM, 38 non-MSM males (MSW, defined as male participants that reported never having sex with men) and 39 females. When examining each subgroup individually, the female group [F= HIV-infected (PWH-F) and HIV seronegative controls (SN-F)]; the men who have sex with men group [MSM= HIV-infected (PWH-MSM) and HIV seronegative controls (SN-MSM)] and the non-MSM male group [MSW= HIV-infected (PWH-MSW) and HIV seronegative controls (SN-MSW)], there were no significant differences in age, BMI, tobacco smoking status, receptive anal intercourse within the past 6 months (RAI), lifetime partners, alcohol intake frequency, or amphetamine use among the PWH and seronegative controls (Supplementary Table 1). All participants were residents of the Netherlands and although some differed in their birth countries, birth countries did not differ significantly between PWH and seronegative controls within each subgroup (Supplementary Table 1). Ninety-one percent of the cohort completed a questionnaire regarding sexual practice during the 6 month period preceding their study visit. Sixteen percent of PWH-F, and ten percent of SN-F declared to have had receptive anal intercourse within the past six months (RAI) (P=0.298 Fisher's exact test). Forty-eight percent of PWH-MSM and sixty-two percent of SN-MSM engaged in RAI (P=0.334 Fisher's exact test, Supplementary Table 1). There were no significant differences in virologic and immunologic characteristics of PWH across subject groups (Supplementary Table 2).
Recruitment	The AGEhIV Cohort Study is an ongoing observational cohort (NCT01466582) of HIV-infected participants from the HIV outpatient clinic of the Amsterdam University Medical Centers (location Academic Medical Center) and HIV-uninfected participants recruited from either the sexual health clinic or the Amsterdam Cohort Studies on HIV/AIDS at the Public Health Service in Amsterdam, the Netherlands. All participants signed informed consent prior to study procedures. Participants were 45 years or older at enrollment and attended biennial study visits. No foreseeable selection biases were observed.
Ethics oversight	Medisch Ethische Toetsingscommissie, Academisch Medisch Centrum, Universiteit van Amsterdam Academisch Medisch Centrum, XT4-140 Meibergdreef 9, 1105 AZ Amsterdam, The Netherlands.

Note that full information on the approval of the study protocol must also be provided in the manuscript.

Clinical data

Policy information about clinical studies

All manuscripts should comply with the ICMJEguidelines for publication of clinical research and a completedCONSORT checklist must be included with all submissions.

Clinical trial registration	NCT01466582, at ClinicalTrials.gov
Study protocol	NCT01466582, at ClinicalTrials.gov
Data collection	Participants were requested to complete a standardized questionnaire concerning demographics, personal and family medical history, use of medications, participation in population screening programs, substance use, quality of life, depression, sexual orientation/behavior/dysfunction, cognitive complaints, calcium/vitamin D intake, physical exercise, social behavior, and work participation/income. All samples and data were collected from 12/15/10 to 5/10/12. For HIV positive participants: the HIV outpatient clinic of the Amsterdam University Medical Centers, location Academic Medical Center was the location of data gathering; for HIV-negative participants, the locations of data gathering were from either the sexual health clinic or the Amsterdam Cohort Studies on HIV/AIDS, both located at Infectious Diseases Research Department of the Public Health Service in Amsterdam, the Netherlands.
Outcomes	Primary clinical outcomes were defined as total comorbidities experienced, which were objectively assessed during each study visit as follows (1) chronic obstructive pulmonary disease (COPD) (GOLD 2 or more by spirometry); (2) advanced liver fibrosis (FIB-4 ≥3.25); (3) decreased kidney function (eGFR <60 mL/min/1.73 m2 using the Chronic Kidney Disease Epidemiology Collaboration [CKD-EPI] equation); (4) osteoporotic fractures (Dual-energy X-ray absorptiometry [DXA] T-score <-2.5 standard deviation (SD) for men aged ≥50 years and post-menopausal women or a Z-score <-2.0 for men aged <50 years and pre-menopausal women using World Health Organization definitions and having reported a fracture 2 years before or after DXA measurement with osteoporosis as an outcome); (5) diabetes mellitus (HbA1c ≥48 mmol/mol or elevated blood glucose (non-fasting ≥11.1 mmol/L or fasting ≥7.0 mmol/L) or using antidiabetic medication). Self-reported comorbidities included: (6) heart failure (diagnosed by cardiologist); (7) non-AIDS defining cancers (confirmed by pathologist, excluding non-melanoma skin cancers); and (8) atherosclerotic disease (diagnosed by specialist; myocardial infarction, angina pectoris, peripheral arterial disease, ischemic stroke or transient ischemic attack).<br>Self-reported diagnoses were validated using hospital records for HIV-positive participants, and general practitioners' (GP) records for HIV-negative participants who had provided consent to contact their GP. Unvalidated diagnoses were only used when validation was not possible (i.e. participants not providing consent to validate self-reported diagnoses with their GP, or documentation about a self-reported diagnosis was absent for HIV-positive participants who received care in other hospitals during follow-up). Of the 35 self-reported comorbidities, 17 (48.6%) were validated as correct, 16 (45.7%) were rejected, and 2 (5.7%) could not be validated.
